# Synergistic anticancer effects of camptothecin and sotorasib in *KRAS*-mutated pancreatic ductal adenocarcinoma

**DOI:** 10.3389/fphar.2025.1635449

**Published:** 2025-07-18

**Authors:** Prasanna Srinivasan Ramalingam, Gayathri Chellasamy, Md Sadique Hussain, Gothandam Kodiveri Muthukaliannan, Tajamul Hussain, Salman Alrokayan, Kyusik Yun, Janaki Ramaiah Mekala, Sivakumar Arumugam

**Affiliations:** ^1^ Protein Engineering lab, School of Biosciences and Technology, Vellore Institute of Technology, Vellore, Tamil Nadu, India; ^2^ Department of Bionanotechnology, Gachon University, Gyeonggi-do, Republic of Korea; ^3^ Uttaranchal Institute of Pharmaceutical Sciences, Uttaranchal University, Dehradun, Uttarakhand, India; ^4^ School of Pharmaceutical Sciences, Lovely Professional University, Phagwara, Punjab, India; ^5^ High Throughput Lab, School of Biosciences and Technology, Vellore Institute of Technology, Vellore, India; ^6^ Centre of Excellence in Biotechnology Research, King Saud University, Riyadh, Saudi Arabia; ^7^ Research Chair for Biomedical Application of Nanomaterials. Biochemistry Department, College of Science, King Saud University, Riyadh, Saudi Arabia; ^8^ School of Biosciences and Technology, Vellore Institute of Technology, Vellore, India

**Keywords:** alkaloid, camptothecin, sotorasib, KRAS mutation, PDAC, synergistic effect

## Abstract

**Background:**

Sotorasib (AMG510) is a first-in-class irreversible, covalent, and selective KRAS G12C inhibitor. However, in patients, acquired clinical resistance was observed within 1 year of its FDA approval. Researchers are exploring combination and repurposing strategies to help overcome this resistance and improve therapeutic efficacy. Several natural compounds have been extensively investigated for their therapeutic potential against various cancers, both individually and in combination with other chemotherapeutic agents. In this study, we examined the synergistic potential of camptothecin and sotorasib in KRAS G12C-mutated MIA PaCa-2 and KRAS G12D-mutated PANC-1 pancreatic ductal adenocarcinoma (PDAC) cells.

**Methods:**

We assessed the half maximal inhibitory concentration (IC50) values of camptothecin and sotorasib using 3-(4,5-dimethylthiazol-2-yl)-2,5-diphenyltetrazolium bromide (MTT) assay, and predicted their synergistic potential using combination index (CI) values and isobologram plots. Proliferation, wound healing, and colony formation assays were performed to examine the chemotherapeutic potential of camptothecin and sotorasib (combination and monotherapy). Reactive oxygen species induction, DNA fragmentation, autophagy flux, and apoptosis and cell cycle analyses were performed using 2′,7′-dichlorofluorescein diacetate (DCFH-DA), 4′,6-diamidino-2-phenylindole (DAPI), LC3-II quantification assays, and flow cytometry analysis. Furthermore, quantitative reverse transcription polymerase chain reaction (qRT-PCR) was performed to analyze gene expression patterns in both pancreatic ductal adenocarcinoma cell lines. Additionally, network pharmacology, gene ontology, and Kyoto Encyclopedia of and Genomes pathway enrichment were performed for camptothecin in PDAC.

**Results:**

The combination therapy with camptothecin and sotorasib resulted in significantly inhibited proliferation, migration, and colony formation; elevated intracellular ROS levels; and induced DNA fragmentation compared with monotherapies in both PDAC cell lines. Flow cytometry and cell cycle analysis revealed that the combination treatment induced apoptosis and G1/S cell cycle arrest. Furthermore, qRT-PCR analysis revealed that the combination therapy significantly upregulated pro-apoptotic genes and downregulated KRAS pathway-related genes, cleaved poly (ADP-ribose) polymerase, anti-apoptotic-related genes as well as autophagy-related genes in both PDAC cell lines. Network pharmacology analysis supports that the identified hub genes play a role in apoptosis and autophagy.

**Conclusion:**

We observed a synergistic relationship between camptothecin and sotorasib in *KRAS*-mutated cancer cells. Furthermore, we recommend examining more natural compounds with chemotherapeutic potential to help overcome clinical resistance of approved chemotherapeutic drugs in the near future.

## 1 Introduction

KRAS mutations at G12, G13, and Q61 positions are predominant and are prevalent in many cancers including pancreatic ductal adenocarcinoma (PDAC), non-small cell lung cancer (NSCLC), and colorectal adenocarcinoma (CRC), and respectively (Prior et al., 2012; Burge and Hobbs, 2022). In general, it acts as a GTPase, and upon mutation, mutant KRAS halts GTP hydrolysis and triggers downstream effector signalling pathways such as MAPK and PI3K-Akt (Conroy et al., 2021). Mutations at G12, G13, and Q61 contribute to nearly 89%, 9%, and 1% of the total mutations, respectively, and G12D and G12C are frequently mutated, contributing to 36% and 23% of all G12 mutations, respectively (Burge and Hobbs, 2022). These KRAS mutants pose significant challenges, such as smooth surface area and lack of druggable pockets, and have been deemed undruggable for the past 3 decades. Several therapeutics such as inhibitors, siRNAs, vaccines, PROTACs, ADCs, and Immunotherapies have been explored against KRAS-mutated cancers (Ramalingam and Arumugam, 2023a; 2023b; Ramalingam et al., 2023a). Notably, after several efforts, sotorasib and adagrasib (MRTX-849) were identified as KRAS G12C inhibitors and approved by the FDA (Skoulidis et al., 2021; Strickler et al., 2023). Following this, several inhibitors have been discovered and studied by employing these compounds as parent molecules, and notably, MRTX1133 is a modified form of MRTX-849 that targets the KRAS G12D mutant (Wang et al., 2022). Furthermore, mutant-selective KRAS inhibitors are also expanding as pan-KRAS inhibitors; for instance, KRAS G12C inhibitors are being studied for KRAS G12D-mutated cancers (Nagasaka et al., 2021).

Sotorasib (AMG510) is the first irreversible, covalent, and selective inhibitor of KRAS G12C-mutated cancers (Lanman et al., 2020). Although sotorasib has been reported to have significant therapeutic potential, recent reports indicate that patients acquire clinical resistance to it (Chan et al., 2023; Mohanty et al., 2023; Ramalingam et al., 2023b). To overcome this limitation, several combination therapies have been investigated in preclinical and clinical trials. For instance, sotorasib in combination with Carboplatin and Pemetrexed (SCARLET study: clinical trial information: jRCT2051210086) indicated a favorable overall response and tolerance rates (Yoshioka et al., 2024). In combination with metformin, it promotes apoptosis and inhibits MAPK and P70S6K respectively (Barrios-Bernal et al., 2023b). Additionally, sotorasib was reported to exhibit reduced cell viability at high concentrations in KRAS G12D PANC-1 cells in combination with MEK inhibitors and irradiation, indicating its potency in combination therapies (Wang et al., 2024). Natural compounds have been explored for their therapeutic potential against various cancers, including PDAC, NSCLC, and CRC (Mishra et al., 2023b; Prajapati et al., 2025; Thumpati et al., 2025). Generally, KRAS-mutated PDAC is highly dependent on autophagy to maintain redox homeostasis and metabolic reprogramming, and its inhibition promotes cell death (Han et al., 2024). Previous reports indicated that DNA damage inducers enhanced the therapeutic efficacy in KRAS-mutated NSCLC, and notably the irinotecan (synthetic camptothecin derivate) showed potential effect against KRAS-mutated CRC and also been used towards metastatic PDAC in clinical settings (Canon et al., 2019; Liu et al., 2022; Formica et al., 2023). Camptothecin is a natural alkaloid isolated from *Camptotheca acuminate*, that is heavily reported for its significant potential to induce apoptotic-mediated cell death in various cancers (Li et al., 2017; Don et al., 2024). It increases the expression of pro-apoptotic genes and decreases the expression of anti-apoptotic genes in PDAC, CRC, and NSCLC (Liskova et al., 2022). Interestingly, in KRAS G12C-mutated MIA PaCa-2 and KRAS G12D-mutated PANC-1, camptothecin has been reported to induce cytotoxicity and DNA damage (Fronza et al., 2011; Ragozin et al., 2018). Camptothecin, in combination with other anticancer agents, has demonstrated synergistic effects in various cancers (Valdez et al., 2022). Based on this, we hypothesized that combining the apoptosis inducer camptothecin could synergize the effect of the KRAS G12C inhibitor sotorasib in KRAS-mutated PDAC and enhance its therapeutic potential.

## 2 Materials and methods

### 2.1 Cell culture and reagents

MIA PaCa-2 harboring the KRAS G12C mutation and the PANC-1 harboring KRAS G12D mutation pancreatic ductal adenocarcinoma cancer cells were purchased from NCCS, India. Both cell lines were cultured in DMEM medium (Gibco) supplemented with 10% FBS (HiMedia) and antibiotic/antimycotic solutions. The cells were properly maintained in 37°C at 5% CO_2_ for further study. Trypsin-EDTA (Gibco), DPBS (Thermo Fisher Scientific), MTT (Sigma), dichlorofluorescein diacetate (Sigma), and DAPI dihydrochloride (Sigma) were purchased. Camptothecin (CPT) and sotorasib were purchased from MedChem Express.

### 2.2 Cell proliferation assay

PDAC cell proliferation was determined using the MTT assay (Mekala et al., 2022). About 1 × 10^5^ cells/mL were seeded in a 96-well plate and maintained in 5% CO_2_ at 37°C for 24 h. Cells were treated with sotorasib and camptothecin to determine their cytotoxic potential against PDAC cells. After the drug treatment, the cells were incubated under the same conditions for 24 h. Following this, the suspension was removed, 20 µL of MTT (5 mg/mL) was added and incubated at 37°C in the dark for 4h. Then, it was solubilized with 100 µL of DMSO and incubated for 5–10 min, and finally, the absorbance was measured at 570 nm in BioTek Epoch microplate reader (Agilent).

### 2.3 Drug synergy analysis

The pharmacodynamic interaction and synergism of sotorasib and camptothecin were determined using Compusyn 1.0 software (Biosoft). It employs the Chou Talay approach to evaluate the combination index (CI) of multiple drug effects (Chou, 2010). CI < 1 denotes synergistic, CI = 1–1.1 denotes additive, and CI > 1.1 denotes antagonistic properties of the compounds. CI was predicted throughout the fractional cell death level range (Fa) (5%–95% death). Notably, for anticancer activity, a CI < 1 with a high effect (Fa) is considered to have greater potential therapeutic effects (Sankar and Muthukaliannan, 2023).

### 2.4 Scratch assay

Both cells were seeded in 6-well plates and when the monolayer reached 90% confluence, 200 µL micropipette tip was used to make the wound, and the sotorasib and camptothecin were added as monotherapy and combination at their respective sub-cytotoxic doses (25% of IC_50_ concentration). Wound healing was observed at various time intervals (0, 24, and 48 h) using an inverted microscope (Labomed TCM-400, United States (Wang et al., 2019).

### 2.5 Colony formation assay

Both cell lines were seeded in 6-well plates (2000 cells/well) and incubated in 5% CO_2_ at 37°C for 24 h Sotorasib and camptothecin were added as monotherapy and in combination at their respective IC_50_ concentrations, and the cells were incubated under the same conditions. Following this, the suspension was removed and the cells were cultured in DMEM at 37°C in 5% CO_2_ for 9–12 days. The cells were then fixed with methanol: acetic acid (3:1) and stained with 0.5% crystal violet, and visible colonies were observed (Nakamura, 2023).

### 2.6 ROS induction assay

The reactive oxygen species levels were determined using a DCFH-DA assay (Eruslanov and Kusmartsev, 2010). Briefly, the cells were seeded in 6-well plates and incubated in 5% CO_2_ at 37°C for 24 h Sotorasib and camptothecin were added as monotherapy and in combination at their respective IC_50_ concentrations, and the cells were incubated under the same conditions. The suspension was then removed and 1 mL of 10 μM DCFH-DA (5 mg/mL) was added to each well and incubated at 37°C for 30 min. The suspension was removed, washed with DMEM and 1× PBS, and the cells were visualized at 485/535 nm (excitation/emission) using a fluorescence microscope (WESWOX FM-3000, India).

### 2.7 DNA fragmentation assay

DNA fragmentation of the cells was determined using the DAPI assay (Chu et al., 2020). Briefly, the cells were seeded in 6-well plates and incubated in 5% CO_2_ at 37°C for 24 h Sotorasib and camptothecin were added as monotherapy and in combination at their respective IC_50_ concentrations, and the cells were incubated under the same conditions. Then the suspension was removed, and 1 mL of 10 μM DAPI was added and incubated for 10 min at 37°C. The suspension was removed, washed with DMEM and 1X PBS, and the cells were visualized at 358/460 nm (excitation/emission) using a fluorescence microscope (WESWOX FM-3000, India).

### 2.8 Apoptosis and cell cycle analysis

Apoptosis and cell cycle analyses were performed using the Annexin V-FITC/PI apoptosis detection kit (Elabscience), according to the manufacturer’s protocol (Wu et al., 2023). Briefly, the cells were seeded in 6-well plates and incubated in 5% CO_2_ at 37°C for 24 h Sotorasib and camptothecin were added as monotherapy and in combination at their respective IC_50_ concentrations, and the cells were incubated under the same conditions. The suspension was removed, the cells were trypsinized, spin at 300 g for 5 min, repeated with PBS, and the supernatant was removed. The cell pellet was resuspended with 500 μL of 1× Annexin V binding buffer, and 5 µL of Annexin V-FITC and PI were added, and incubated at room temperature for 15–20 min in dark. The cells were counted at 518/620 nm (Annexin V/PI) using a flow cytometer (CytoFLEX, Beckman Coulter, United States). For cell cycle analysis, similar protocol was followed and 5 µL of PI alone was added and the cells were counted at 620 nm (Solanki et al., 2024).

### 2.9 Autophagy analysis by MAP1LC3B quantification

LC3II levels in cells were quantified using the MAP1LC3B ELISA kit (CUSABIO) according to the manufacturer’s protocol (Longobardi et al., 2024). Briefly, cells were seeded and incubated in 5% CO_2_ at 37°C for 24 h Sotorasib and camptothecin were added as monotherapy and in combination at their respective IC_50_ concentrations, and the cells were incubated under the same conditions. Then the suspension was removed, 100 μL of Biotin-antibody was added, incubated at 37°C for 60 min, followed by aspiration and wash, to which 100 μL of HRP-avidin was added, incubated at 37°C for 60 min. It was again aspirated and washed, and 90 μL of TMB substrate was added, incubated at 37°C for 30 min in the dark. Then, 50 μL of stop solution was added, and the absorbance was quantified at 450 nm in the BioTek Epoch microplate reader (Agilent).

### 2.10 RNA isolation and qRT-PCR analysis

Sotorasib and camptothecin were added as monotherapy and in combination at their respective IC_50_ concentrations for 24 h, and RNA was isolated using TRIzol reagent (Takara) according to the manufacturer’s protocol. The isolated RNA was quantified (at 260 nm) and the cDNA strand was synthesized using the PrimeScript RT reagent kit (Takara) according to the manufacturer’s protocol. Then the gene expression was performed in CFX96 Touch Real-Time PCR Detection System, BioRad using the TB Green^®^ Advantage^®^ qPCR Premix (Takara) as per the manufacturer’s protocol. GAPDH was used as a control, and the sequences of all primers used in this study are listed in Table 1. The mRNA expression levels were determined by 2^−ΔΔCT^ method, and the data were normalized to GAPDH, and all the reactions were carried out in triplicates to ensure the reliability of the gene expression levels (Singh et al., 2017; Suhail et al., 2022).

**TABLE 1:** List of primers used in the study.

Gene name	Forward primer (5′-3′)	Reverse primer (5′-3′)	Product length (bp)
GAPDH	GGT​GAA​GGT​CGG​TGT​GAA​CG	CTC​GCT​CCT​GGA​AGA​TGG​TG	232
BAX	GCC​CTT​TTG​CTT​CAG​GGT​TTC	GCA​GGG​TAG​ATG​AAT​CGG​GG	613
BID	GGTGCAGGCCACCCTTG	CCG​ACT​CAC​TCC​TGG​TTC​AC	421
BAK	GCAGGCTGATCCCGTCC	GGC​TAA​GGA​GGT​CCC​AGA​GA	935
BCL-2	GAA​CTG​GGG​GAG​GAT​TGT​GG	CAT​CCC​AGC​CTC​CGT​TAT​CC	164
BCL-XL	CGG​ATT​TGA​ATC​TCT​TTC​TCT​CCC	CGA​CCC​CAG​TTT​ACC​CCA​TC	557
PARP	CTC​AGG​GGA​GGG​TCT​GAT​GA	CTT​TGA​CAC​TGT​GCT​TGC​CC	517
KRAS	TAC​ATG​AGG​ACT​GGG​GAG​GG	AGG​CAT​CAT​CAA​CAC​CCA​GA	380
BRAF	ATT​CCG​GAG​GAG​GTG​TGG​A	TCT​CTG​CTA​AGG​ACG​CCT​CT	802
MEK	GGC​AAC​AGG​ACA​GTT​TCC​CT	TCC​GTT​CAC​AGT​GTC​TGT​CG	554
ERK	ACT​CCA​AAG​CCC​TTG​ACC​TG	CTT​CAG​CCG​CTC​CTT​AGG​TA	185
ATG5	TGC​AGA​TGG​ACA​GTT​GCA​CA	CCA​CTG​CAG​AGG​TGT​TTC​CA	139
MAP1LC3B	GGTTCACAAAACCCGCCG	AGT​CAG​GGC​CGT​TTT​CTC​AC	173

### 2.11 Statistical analysis

All statistical tests were performed using GraphPad Prism version 8. The data collected from different experiments were analyzed using ANOVA and Dennett’s multiple comparisons test. All the experiments were executed at least in triplicates (n = 3), and represented in mean ± SD. Statistical significance was set at P < 0.05. * indicates P < 0.05, ** indicates P < 0.01, *** indicates P < 0.005, **** indicates P < 0.001 respectively.

### 2.12 Network pharmacology analysis

The potential targets of sotorasib (Soto), camptothecin (camp), and pancreatic ductal adenocarcinoma (PDAC) were retrieved from ChEMBL (https://www.ebi.ac.uk/chembl/), Swisstargetprediction (http://www.swisstargetprediction.ch/), and GeneCards (https://www.genecards.org/), respectively (Safran et al., 2010; Daina et al., 2019; Zdrazil et al., 2024). The commonly shared genes between PDAC, sotorasib, and camptothecin were predicted using Venny 2.1 (https://bioinfogp.cnb.csic.es/tools/venny/), and their protein-protein interaction (PPI) network was identified using STRING (https://string-db.org/) (Szklarczyk et al., 2023). The Cytohubba plugin was used to identify the top hub genes using parameters including MCC, degree, betweenness, and closeness in Cytoscape (Kohl et al., 2011). GO and KEGG pathway enrichment analyses were performed using the ShinyGO (https://bioinformatics.sdstate.edu/go/) and Enrichr webservers (https://maayanlab.cloud/Enrichr/), respectively (Kuleshov et al., 2016; Ge et al., 2020).

## 3 Results

### 3.1 Camptothecin increases sotorasib-induced cytotoxicity in KRAS-mutated PDAC cells

**FIGURE 1:**
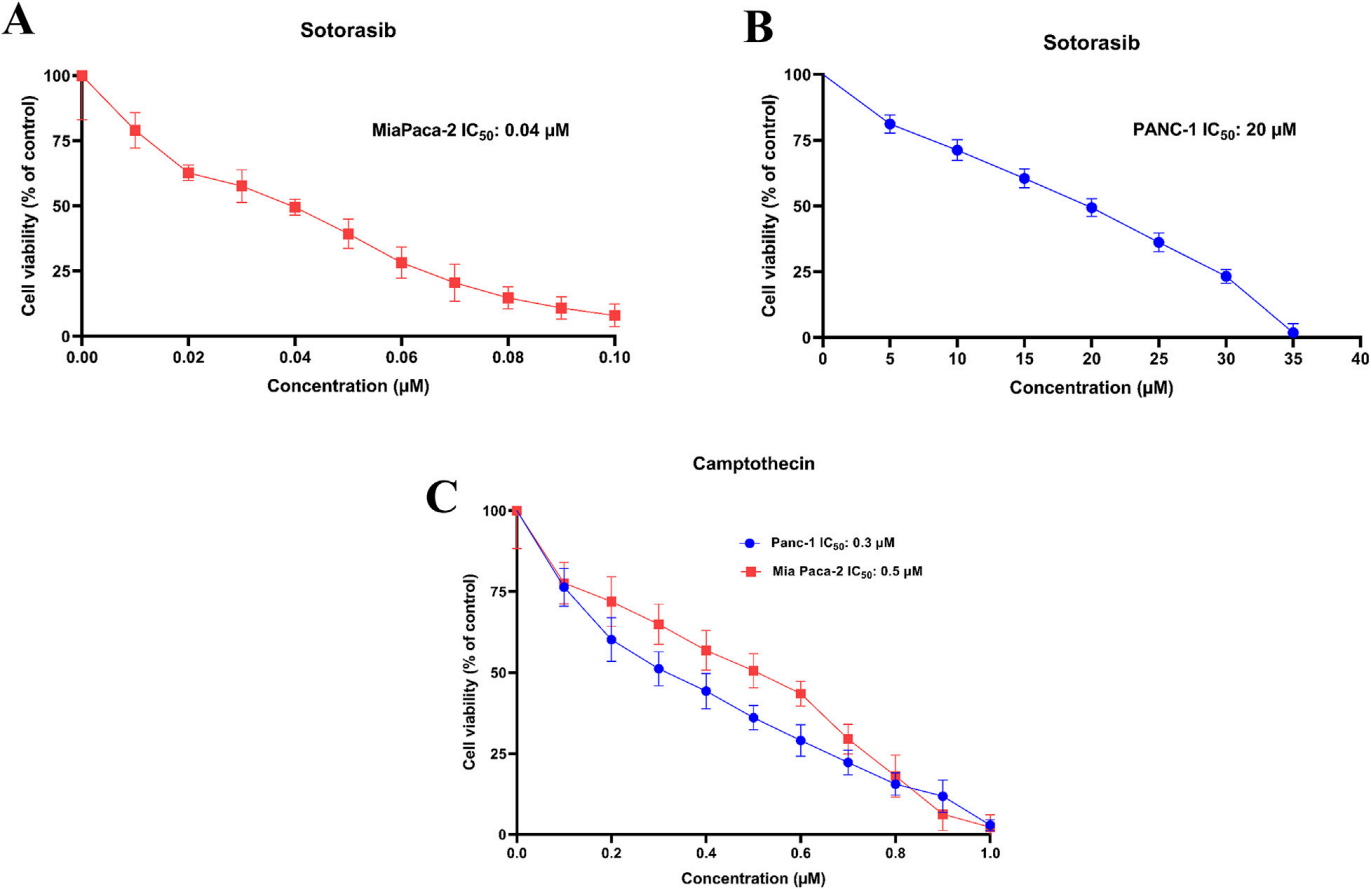
IC_50_ plots of sotorasib in MIA PaCa-2 **(A)** and PANC-1 **(B)** cells and IC_50_ plots of camptothecin MIA PaCa-2 and PANC-1 cells **(C)**. The viability percentage was calculated for 24h and normalized to the control**.** All the experiments were performed in double triplicates (n = 6) and the data were shown as mean ± SD respectively.

**FIGURE 2:**
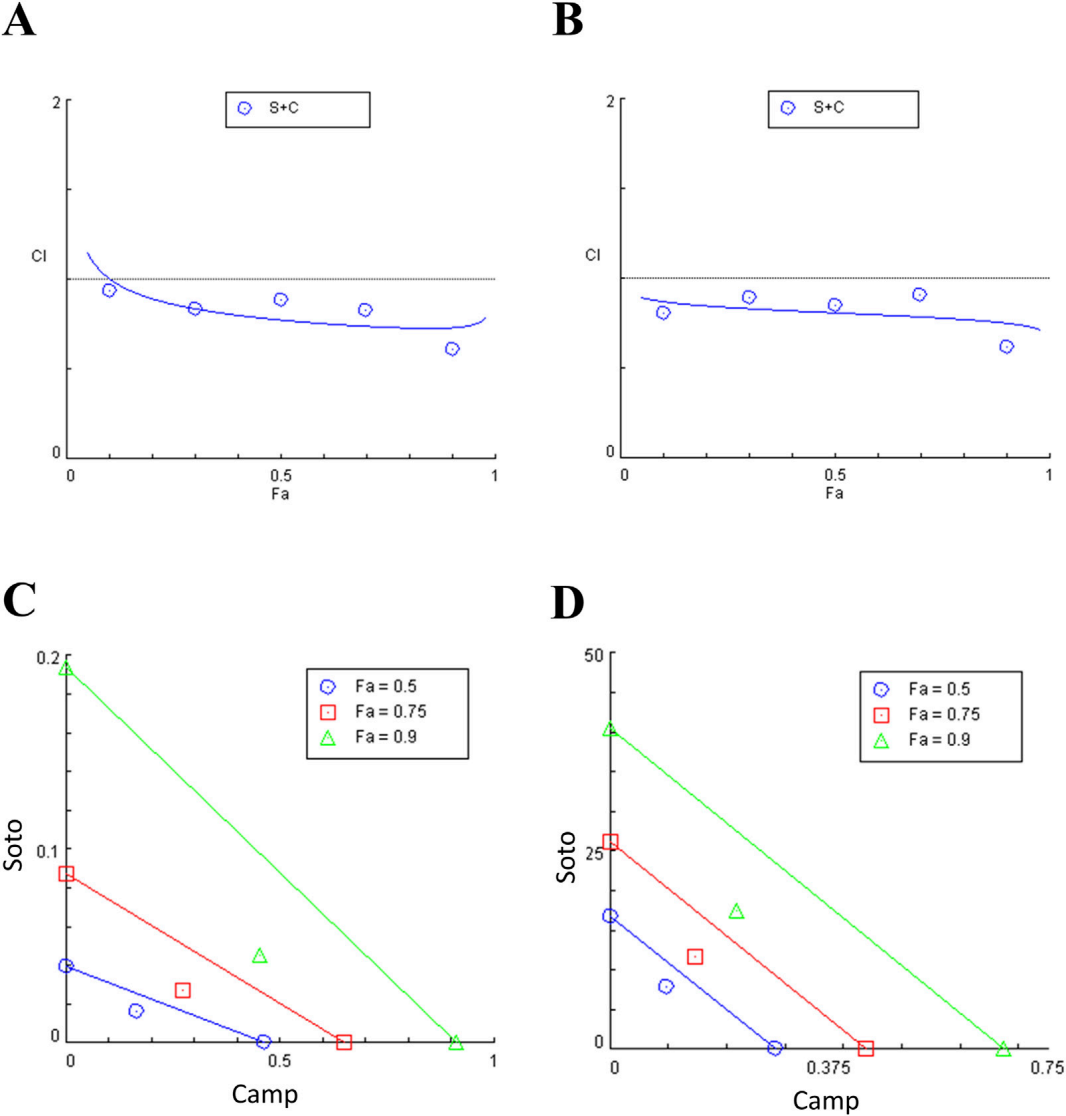
Combination index (CI) plots and Isobologram of sotorasib + camptothecin combination in MIA PaCa-2 **(A and C)** and PANC-1 **(B and D)** cells. All the experiments were performed in double triplicates (n = 6) and the data were shown as mean ± SD respectively.

Cytotoxic potential of sotorasib and camptothecin in KRAS-mutated PDAC cells were determined. The IC_50_ of sotorasib in MIA PaCa-2 and PANC-1 was determined as 0.04 μM and 20 μM, and the IC_50_ of camptothecin in MIA PaCa-2 and PANC-1 was determined as 0.5 μM and 0.3 μM respectively as shown in Figure 1. The compounds exhibited potential cytotoxicity towards KRAS-mutated cells in a dose-dependent manner. The synergistic nature of camptothecin with sotorasib was determined using Compsyn, and the CI plots and isobologram plots as shown in Figure 2. In MIA PaCa-2 cells, the sotorasib + camptothecin combination showed a CI < 1 at all combination points (0.1, 0.3, 0.5, 0.7, and 0.9), and high Fa (high activity) showed a CI = 0.615. Similarly, in PANC-1 cells, the sotorasib + camptothecin combination showed CI < 1 at all combination points (0.1, 0.3, 0.5, 0.7, and 0.9), and high Fa (high activity) showed CI = 0.621, indicating that their combination had a greater therapeutic effect. In addition, in the isobologram plots of both cells, the combination points at Fa = 0.5, 0.75, 0.9 lies to the lower left of the additive line, indicating synergism, as shown in Figure 2.

### 3.2 Camptothecin and sotorasib combination potentially inhibits proliferation, migration, and colony formation

**FIGURE 3:**
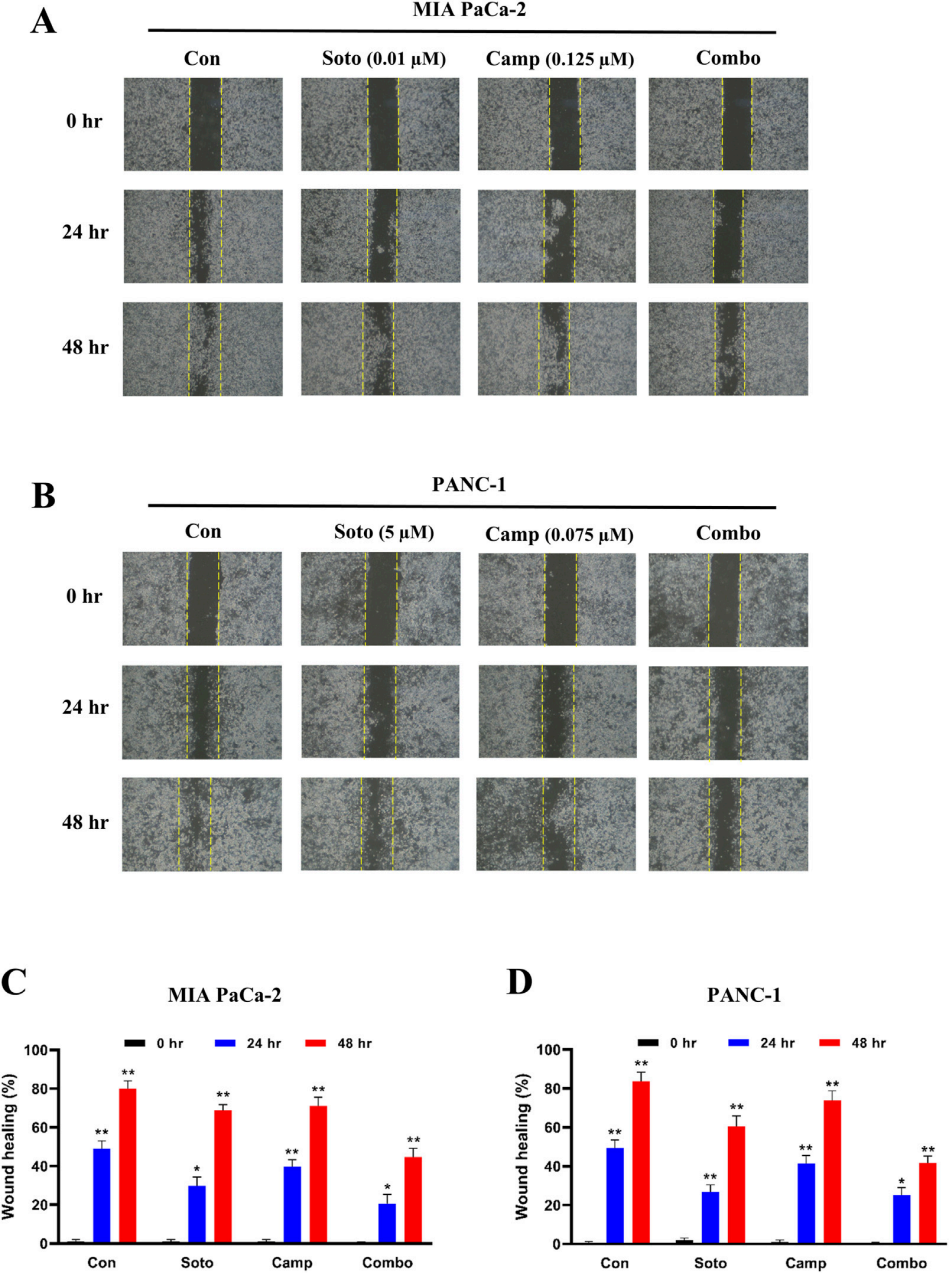
Wound healing of sotorasib and camptothecin monotherapy and their combination in MIA PaCa-2 **(A and C)** and PANC-1 **(B and D)** cells. Sotorasib at 0.01 µM and 5 µM were treated in MIA PaCa-2 and PANC-1 cells; and camptothecin at 0.125 µM and 0.075 µM were treated in MIA PaCa-2 and PANC-1 cells respectively. The wound closure was measured at 0, 24, and 45 h. All the experiments were performed in triplicates (n = 3) and the data were shown as mean ± SD respectively.

**FIGURE 4:**
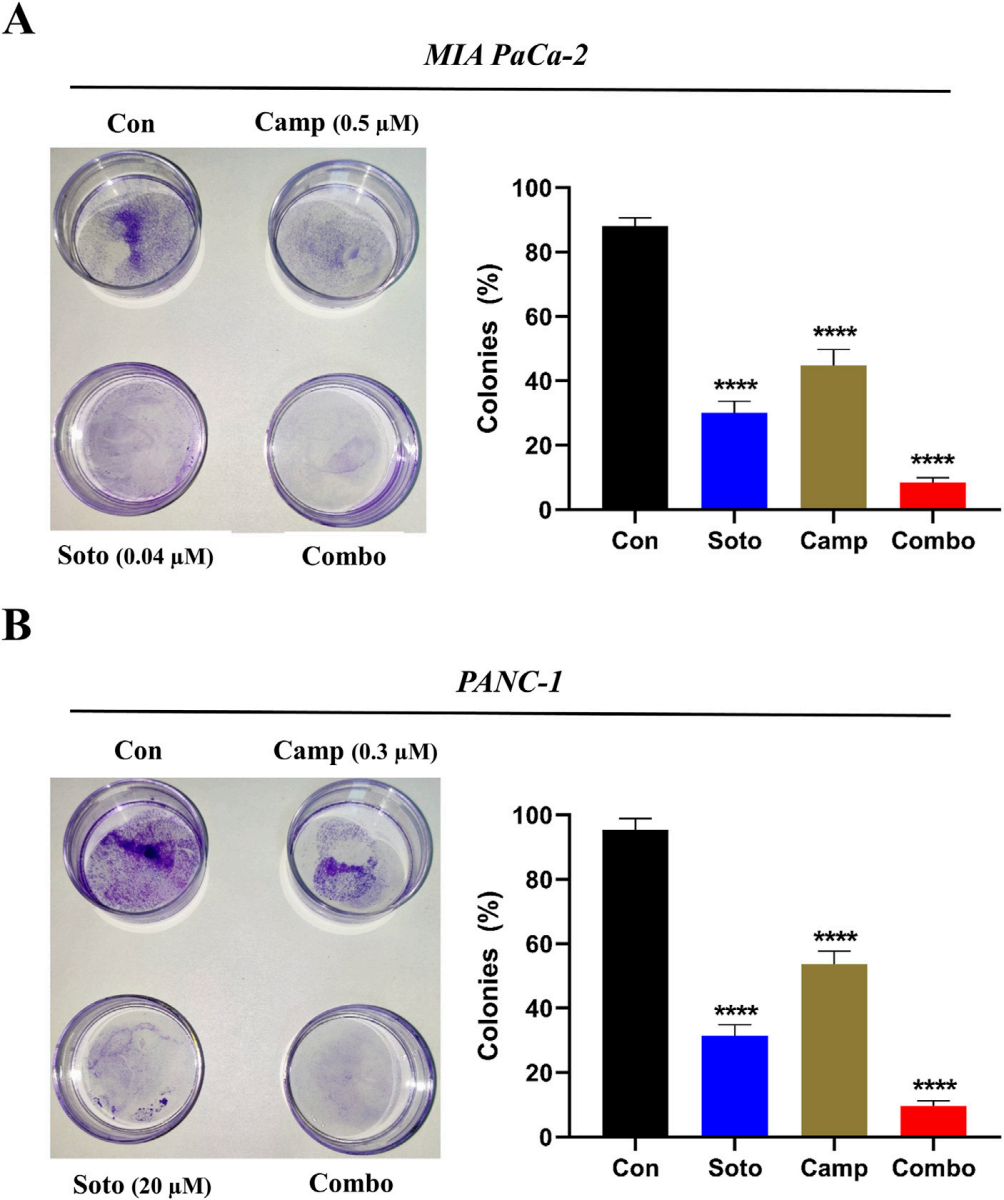
Colony formation of sotorasib and camptothecin monotherapy and their combination in MIA PaCa-2 **(A)** and PANC-1 **(B)** cells. Sotorasib at 0.04 µM and 20 µM were treated in MIA PaCa-2 and PANC-1 cells; and camptothecin at 0.5 µM and 0.3 µM were treated in MIA PaCa-2 and PANC-1 cells respectively. All the experiments were performed in triplicates (n = 3) and the data were shown as mean ± SD respectively.

After examining their synergistic effects, camptothecin and sotorasib were tested for their roles in the migration of KRAS-mutated PDAC cells using a scratch assay. In MIA PaCa-2, after 24 h, the wound closure of sotorasib, camptothecin, and sotorasib + camptothecin combination was observed to be 30%, 40%, and 21%, respectively, and after 48 h, it was 69%, 73%, and 45%, respectively. In PANC-1 cells, after 24 h, the wound closure was observed to be 27%, 41%, and 25%, and after 48 h, it was 60%, 74%, and 42% for sotorasib, camptothecin, sotorasib + camptothecin, respectively, as shown in Figure 3. Notably, in both cells, the compounds in both monotherapy and combination exhibited anti-migration potential compared with the control group in a time-dependent manner. In addition, the combination showed greater potential than the individual treatments, indicating its significant potential in inhibiting the migration of KRAS-mutated PDAC cells. In addition, a clonogenic assay indicated that colony formation was inhibited in both cells. In MIA PaCa-2, sotorasib, camptothecin, and sotorasib + camptothecin combination showed 30%, 45%, and 9% colony formation, and in PANC-1 cells, it showed 32%, 54%, and 10% colony formation, respectively. Comparatively, the drug-treated groups showed significantly reduced colony formation. Notably, the combination showed greater potential than the monotherapy treatment groups, as shown in Figure 4.

### 3.3 Camptothecin combined with sotorasib modulates the ROS production and DNA fragmentation

**FIGURE 5:**
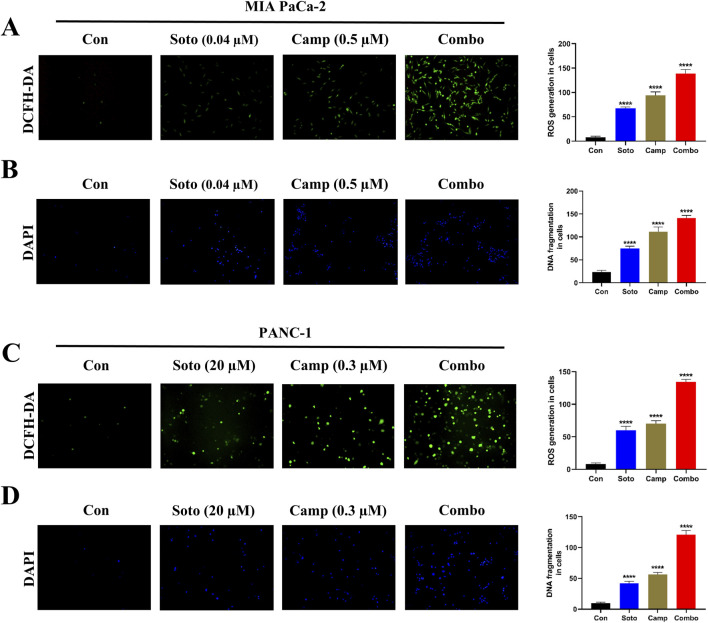
Intracellular ROS production (DCFH-DA) and DNA fragmentation (DAPI) of sotorasib and camptothecin monotherapy and their combination. DCFH-DA **(A)** and DAPI **(B)** assays of drug treatment in MIA PaCa-2 cells, and DCFH-DA **(C)** and DAPI **(D)** assays of drug treatment in PANC-1 cells. Sotorasib at 0.04 µM and 20 µM were treated in MIA PaCa-2 and PANC-1 cells; and camptothecin at 0.5 µM and 0.3 µM were treated in MIA PaCa-2 and PANC-1 cells respectively. All the experiments were performed in triplicates (n = 3) and the data were shown as mean ± SD respectively.

Intracellular ROS production and DNA fragmentation in drug-treated cells were determined using DCFH-DA and DAPI assays, respectively. In MIA PaCa-2 and PANC-1 cells, the sotorasib, camptothecin, and sotorasib + camptothecin elevated ROS levels by 8.4 fold, 11.8 fold and 16.8 fold and 7.3 fold, 8.6 fold and 16.3 fold higher than the control, respectively, as shown in Figure 5. Additionally, In MIA PaCa-2 and PANC-1 cells, the combinations of sotorasib, camptothecin, and sotorasib + camptothecin induced DNA fragmentation by 4.2 fold, 6.2 fold and 7.8 fold and 4.2 fold, 4.6 fold and 12.2 fold higher than the control, as shown in Figure 5. From the results, it was observed that the sotorasib + camptothecin combination had more potential than their monotherapy in inducing ROS levels and DNA fragmentation in both cell lines. In addition, higher activity was observed in KRAS G12C-mutated MIA PaCa-2 cells than in KRAS G12D-mutated PANC-1 cells. Elevated ROS levels and DNA fragmentation in cancer cells are also associated with apoptosis and autophagy, indicating that this drug combination can induce apoptosis- or autophagy-mediated cell death in KRAS-mutated PDAC.

### 3.4 Camptothecin promotes sotorasib-induced apoptosis and induces cell cycle arrest

**FIGURE 6:**
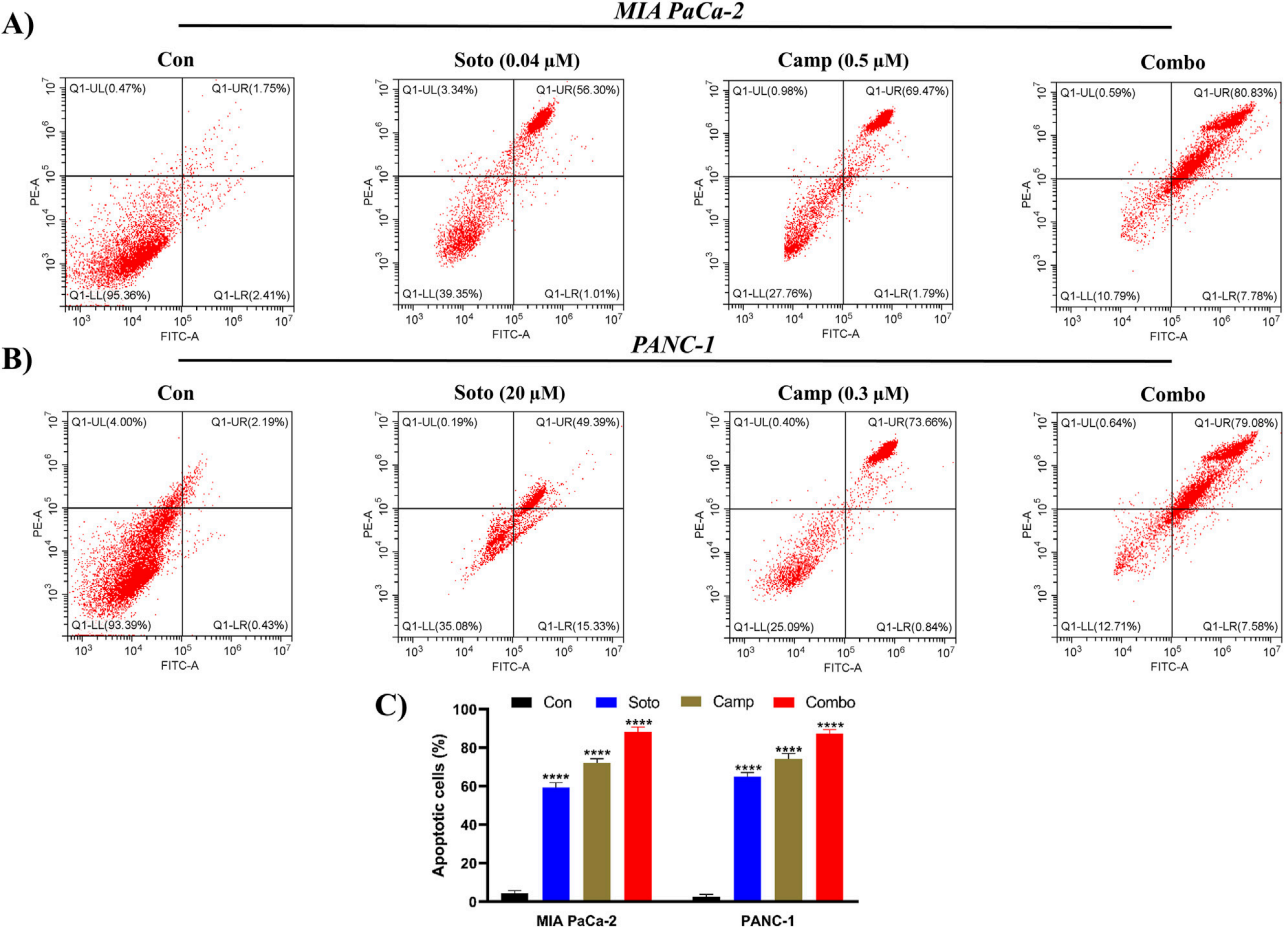
Apoptosis detection by Annexin FITC/PI in flow cytometry in MIA PaCa-2 **(A and C)** and PANC-1 cells **(B and C)**. The apoptosis induced cells in the control and treated groups were determined by employing FITC detection in x-axis and PI detection in y-axis respectively. In the plot, LL, LR, UR, and UL represents the amount of cells in the normal, early apoptotic, late apoptotic, and necrosis states respectively. Sotorasib at 0.04 µM and 20 µM were treated in MIA PaCa-2 and PANC-1 cells; and camptothecin at 0.5 µM and 0.3 µM were treated in MIA PaCa-2 and PANC-1 cells respectively. All the experiments were performed in triplicates (n = 3) and the data were shown as mean ± SD respectively.

**FIGURE 7:**
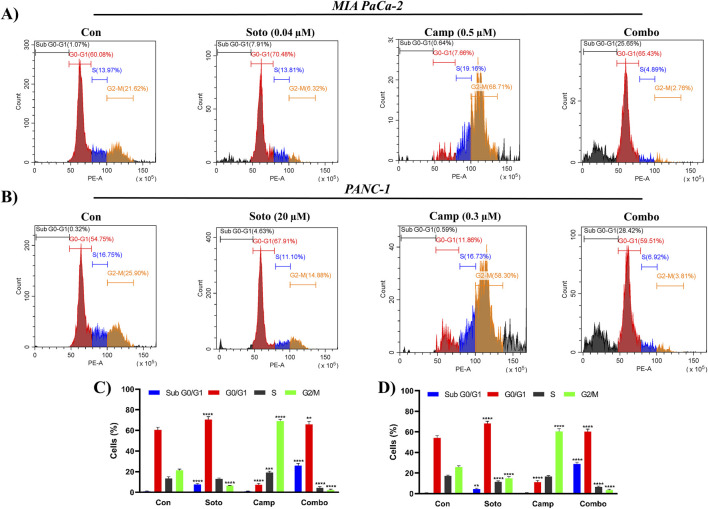
Cell cycle analysis by PI in flow cytometry in MIA PaCa-2 **(A and C)** and PANC-1 cells **(B and D)**. The Sub G1, G0/G1, S and G2/M phases are highlighted in blue, red, brown and green color respectively. Sotorasib at 0.04 µM and 20 µM were treated in MIA PaCa-2 and PANC-1 cells; and camptothecin at 0.5 µM and 0.3 µM were treated in MIA PaCa-2 and PANC-1 cells respectively. All the experiments were performed in triplicates (n = 3) and the data were shown as mean ± SD respectively.

The apoptosis induction potential of drug-treated cells was determined using flow cytometry. In MIA PaCa-2 cells, the combination of sotorasib, camptothecin, and sotorasib + camptothecin induced apoptosis in 57.31%, 71.26%, and 88.61% respectively, as shown in Figure 6. Similarly, in PANC-1 cells, apoptosis was observed in 64.72%, 74.5%, and 86.66% of cells treated with sotorasib, camptothecin, and sotorasib + camptothecin combination, respectively. Notably, camptothecin is a known apoptotic inducer that potentially induces more apoptosis than sotarasib-treated cells; however, the combination of camptothecin and sotarasib induced more apoptosis than monotherapy alone. Additionally, the compounds specifically induced apoptosis in KRAS G12C and KRAS G12D-mutated cells, and no considerable necrosis was observed in any of the treatment groups, as depicted in Figure 6. In cell cycle analysis, we observed that in both cells, sotorasib induced G1/S phase arrest, camptothecin induced G2/M phase arrest, and the sotorasib + camptothecin combination induced G1/S phase arrest, with elevated Sub G0/G1 phase, indicating apoptosis induction. Combination treatment resulted in 25.65% and 28/.42% in the G0/G1 phase of MIA PaCa-2 and PANC-1 cells, respectively. In addition, cell cycle analysis revealed that the sotorasib + camptothecin combination had more apoptosis induction potential than their monotherapies by increasing the Sub G0 phases and inducing G1/S phase arrest in both PDAC cell lines, as shown in Figure 7.

### 3.5 Camptothecin and sotorasib combination regulates autophagy in PDAC cells

**FIGURE 8:**
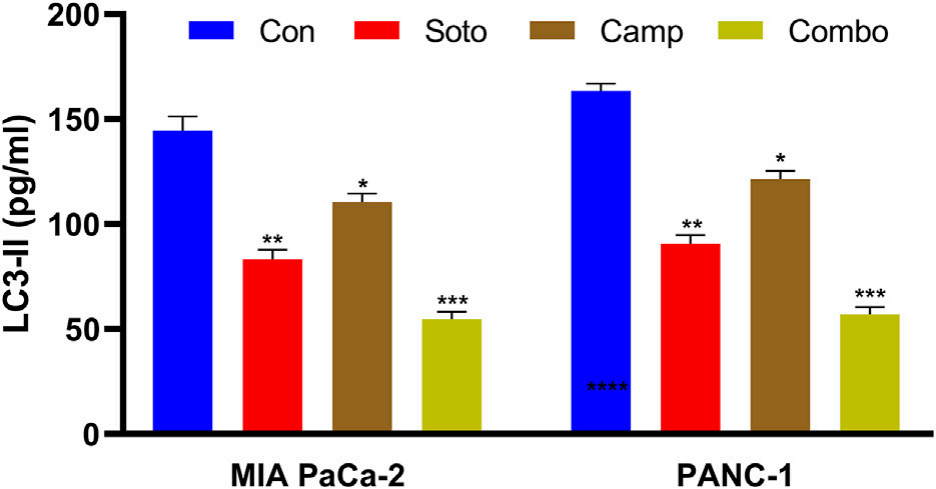
LC3-II quantification in MIA PaCa-2 and PANC-1 cells. Sotorasib at 0.04 µM and 20 µM were treated in MIA PaCa-2 and PANC-1 cells; and camptothecin at 0.5 µM and 0.3 µM were treated in MIA PaCa-2 and PANC-1 cells respectively. All the experiments were performed in triplicates (n = 3) and the data were shown as mean ± SD respectively.

Generally, KRAS-mutated PDAC are highly dependent on autophagy, which is promoted to maintain redox homeostasis and metabolic reprogramming. LC3-II (the lipidated form of MAP1LC3B), which belongs to the LC3 system, is involved in autophagosome formation and commonly used to observe autophagic flux. In our study, we quantified LC3-II levels after drug treatment in both cell lines. Initially, the MAP1LC3B standard curve was plotted as shown in Supplementary Figure S1 (Supplementary Material S1), and the data were interpolated for the observed OD values. The sotorasib, camptothecin, sotorasib + camptothecin combination showed around 82.12 pg/mL, 111.89 pg/mL, 53.62 pg/mL in MIA PaCa-2 and around 89.05 pg/mL, 119.83 pg/mL, 60.57 pg/mL in PANC-1 cells respectively as shown in Figure 8. This indicates that the sotorasib + camptothecin combination significantly inhibits autophagy compared to the monotherapies, reduces the dependency of mutated KRAS PDAC on autophagy, and leads to effective treatment.

### 3.6 Camptothecin and sotorasib combination regulates the expression of MAPK, apoptotic and autophagy-related genes

**FIGURE 9:**
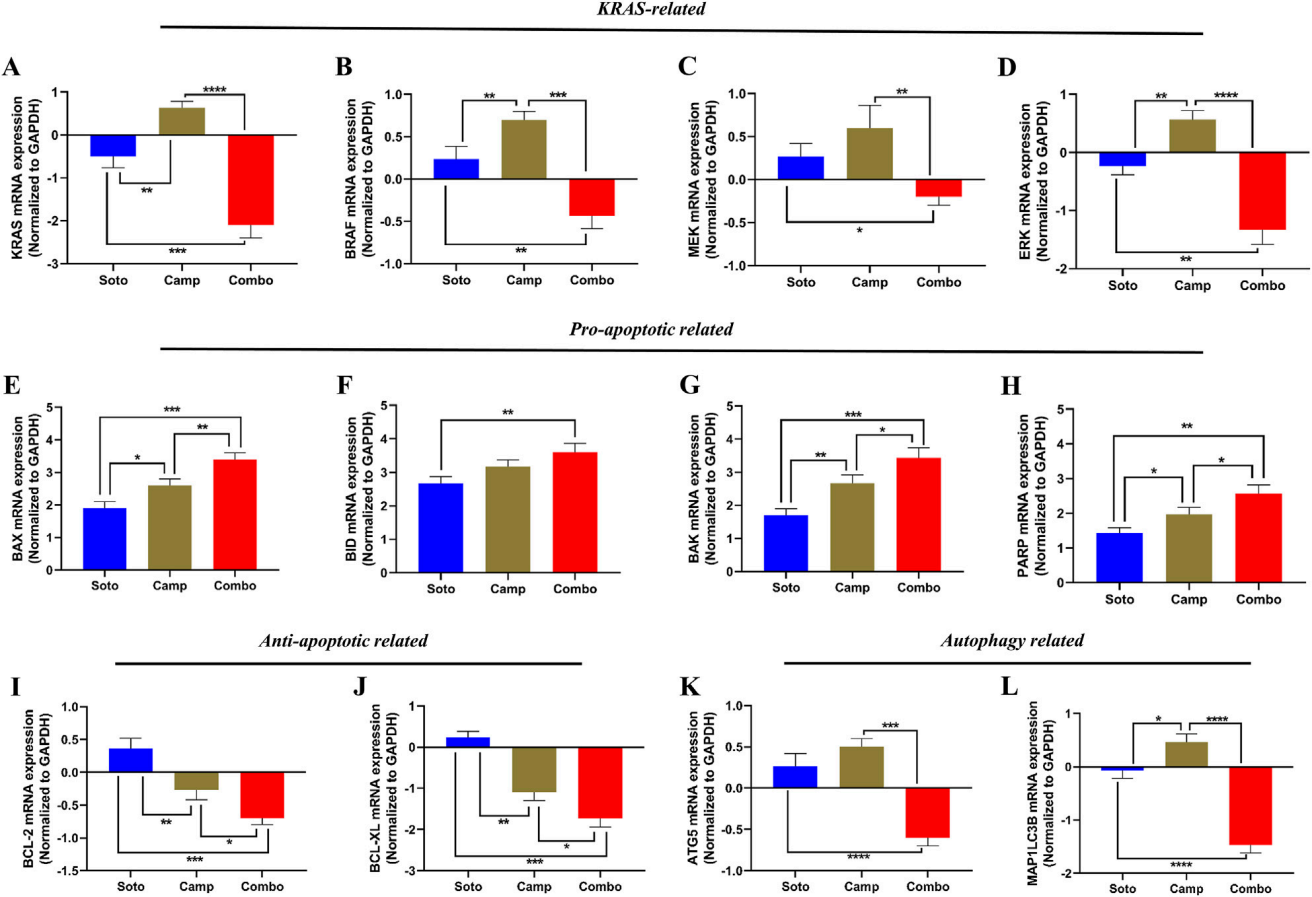
mRNA expression analysis of KRAS related, pro-apoptotic and anti-apoptotic related genes in sotorasib and camptothecin treated MIA PaCa-2 cells. All the mRNA expression levels were normalized with GAPDH, and the normalized expression levels of KRAS **(A)**, BRAF **(B)**, MEK **(C)**, ERK **(D)**, BAX **(E)**, BID **(F)**, BAK **(G)**, PARP **(H)**, BCL-2 **(I)**, BCL-XL **(J),** ATG5 **(K)**, and MAP1LC3B **(L)** were calculated by 2^−ΔΔCT^ method. Sotorasib at 0.04 µM and 20 µM were treated in MIA PaCa-2 and PANC-1 cells; and camptothecin at 0.5 µM and 0.3 µM were treated in MIA PaCa-2 and PANC-1 cells respectively. All the experiments were performed in triplicates (n = 3) and the data were shown as mean ± SD respectively.

**FIGURE 10:**
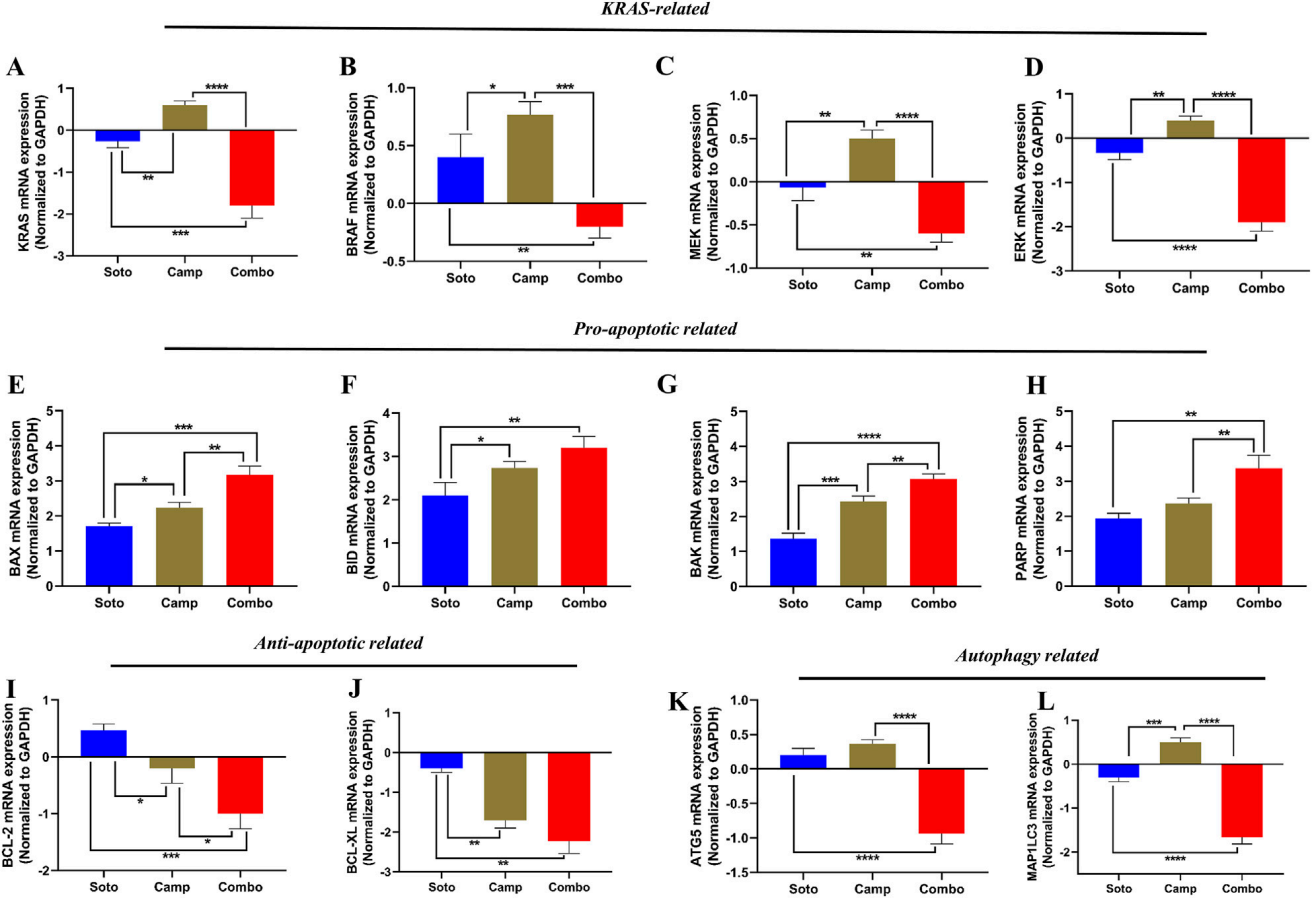
mRNA expression analysis of KRAS related, pro-apoptotic and anti-apoptotic related genes in sotorasib and camptothecin treated PANC-1 cells. All the mRNA expression levels were normalized with GAPDH, and the normalized expression levels of KRAS **(A)**, BRAF **(B)**, MEK **(C)**, ERK **(D)**, BAX **(E)**, BID **(F)**, BAK **(G)**, PARP **(H)**, BCL-2 **(I)**, BCL-XL **(J),** ATG5 **(K)**, and MAP1LC3B **(L)** were calculated by 2^−ΔΔCT^ method. Sotorasib at 0.04 µM and 20 µM were treated in MIA PaCa-2 and PANC-1 cells; and camptothecin at 0.5 µM and 0.3 µM were treated in MIA PaCa-2 and PANC-1 cells respectively. All the experiments were performed in triplicates (n = 3) and the data were shown as mean ± SD respectively.

The role of the sotorasib and camptothecin combination and their monotherapies in the expression of KRAS pathway-related genes (KRAS, BRAF, MEK, and ERK), proapoptotic-related genes (BAX, BID, and BAK), cleaved PARP, antiapoptotic-related genes (BCL-2 and BCL-XL), and autophagy-related genes (ATG5 and MAP1LC3B) were analyzed by qRT-PCR. The mRNA expression levels were determined by 2^−ΔΔCT^ method, and the data were normalized to GAPDH. The combination of sotorasib and camptothecin potentially regulated gene expression compared to monotherapy in both PDAC cell lines. Interestingly, the combination of sotorasib and camptothecin significantly upregulated pro-apoptotic genes and downregulated KRAS pathway, anti-apoptotic, and autophagy genes in both cell lines, as depicted in Figures 9, 10. In MIA PaCa-2 cells, the combination treatment significantly upregulated the pro-apoptotic genes BAX (3.5-fold increase), BID (3.8-fold increase), BAK (3.8-fold increase), and PARP (3-fold increase) and downregulated the pro-apoptotic genes BCL-2 (0.7-fold decrease) and BCL-XL (2-fold decrease). Similarly, in PANC-1 cells, the combination treatment significantly upregulated the pro-apoptotic genes BAX (3.5-fold increase), BID (3.5-fold increase), BAK (3.2-fold increase), and PARP (3.8-fold increase) and downregulated the pro-apoptotic genes BCL-2 (1.2-fold decrease) and BCL-XL (2.5-fold decrease). In the context of autophagy, combination treatment significantly downregulated the autophagy genes ATG5 (1.7-fold decrease) and MAP1LC3B (2.5-fold decrease) in MIA PaCa-2 cells, and ATG5 (2.1-fold decrease) and MAP1LC3B (2.8-fold decrease) in PANC-1 cells. Notably, in both KRAS G12C-mutated and KRAS G12D-mutated cells, sotorasib and camptothecin combination treatment significantly downregulated KRAS expression by 2.4-fold and 2-fold, respectively. In addition, the combination treatment downregulated KRAS downstream effectors such as BRAF, MEK, and ERK in both PDAC cell lines, indicating that it inhibits KRAS-related genes and promotes apoptosis while suppressing autophagy by modulating gene expression levels. Overall, the heat map illustrating the fold changes in gene expression obtained from RT-PCR analysis was shown in Supplementary Figure S2 (Supplementary Material S1).

### 3.7 Network pharmacology analysis highlights the significance of camptothecin and sotorasib in apoptosis and autophagy regulation

**FIGURE 11:**
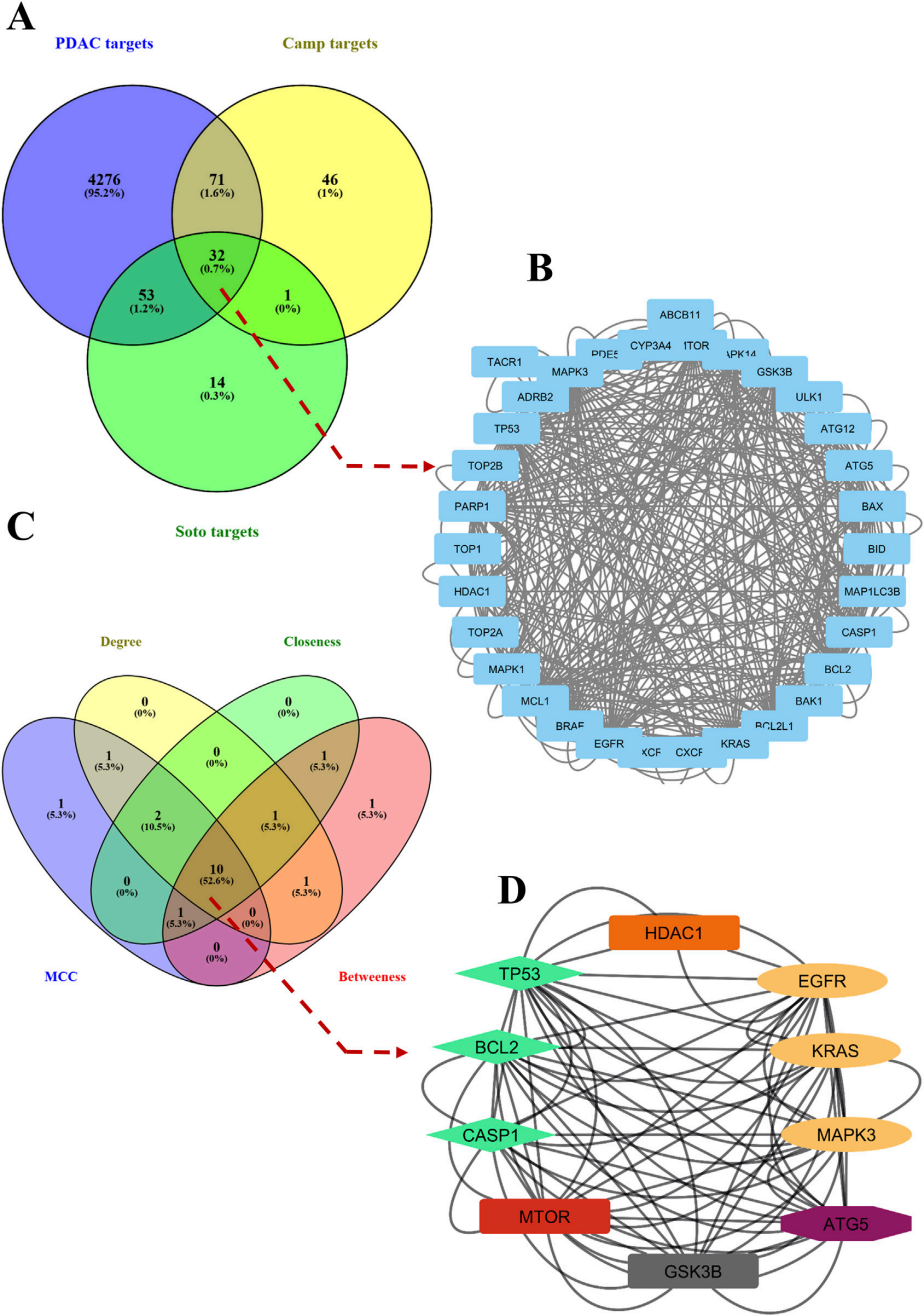
Commonly shared targets of pancreatic ductal adenocarcinoma (PDAC), sotorasib (Soto) and camptothecin (Camp) were showed in Venn diagram **(A)**. The protein-protein interaction (PPI) network of 32 commonly shared targets were showed **(B)**. Then the hub genes were identified by using parametes like MCC, degree, betweenness, and closeness and showed in Venn diagram **(C)**. The PPI interaction network of 10 hub genes were shown, in which the apoptosis related genes (BCl-2 and CASP1), KRAS pathway (EGFR, KRAS, MAPK3), and apoptosis related (ATG5) were shown in green, yellow and purple color respectively **(D)**.

**FIGURE 12:**
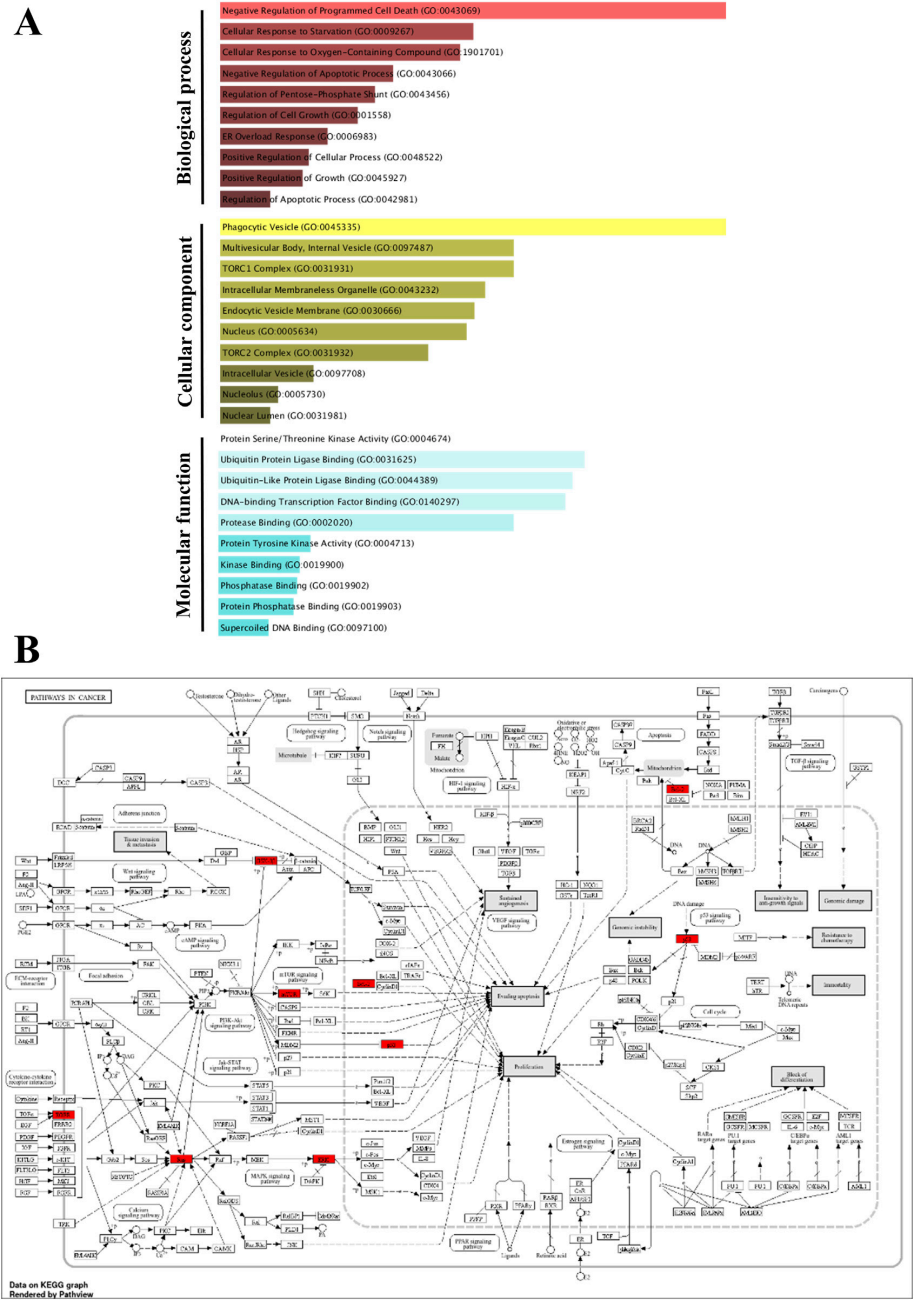
Gene Ontology (GO) and KEGG pathway enrichment of the identified hub genes. The GO enriched terms like biological process (BP), cellular component (CC) and molecular function (MF) were shown in p-value ranking **(A)**. The KEGG of pathways in cancer indicates the hub genes in red color **(B)**.

**FIGURE 13:**
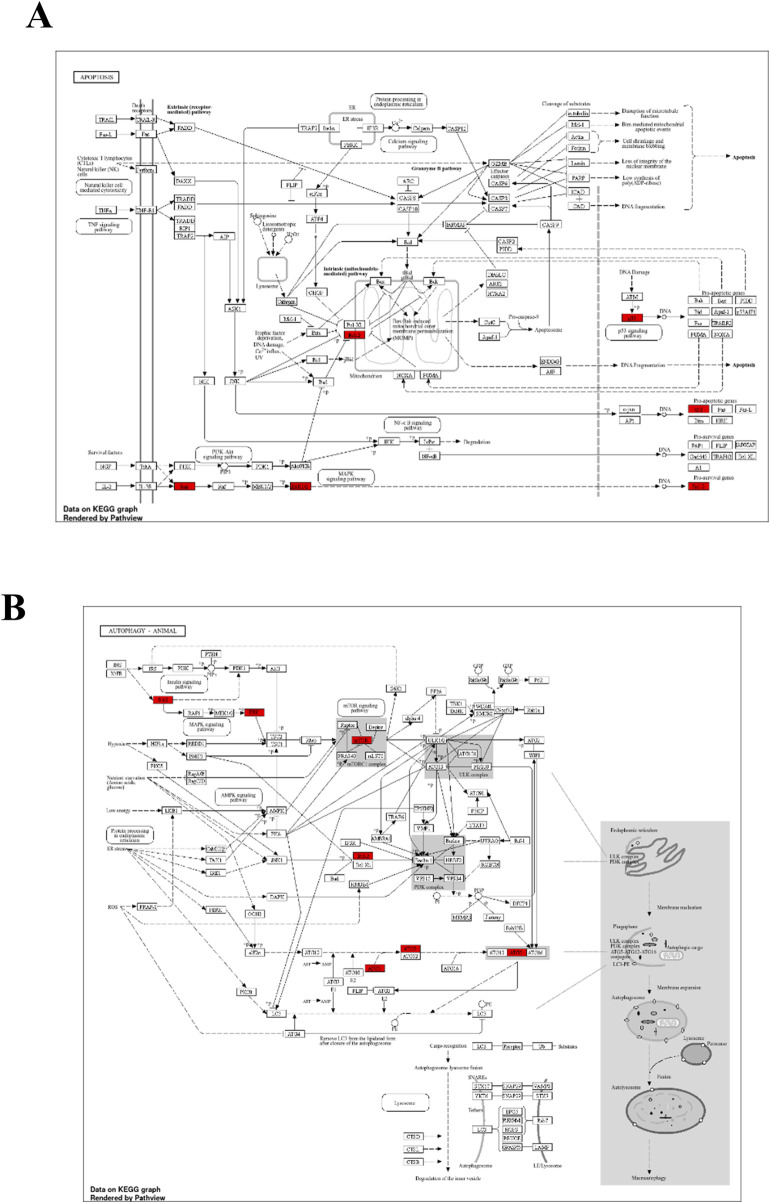
The KEGG of Apoptosis **(A)** and Autophagy **(B)** indicates the hub genes in red color.

To further support the significance of the combination of camptothecin and sotorasib in the regulation of apoptosis and autophagy, network pharmacology analysis was performed. The probable potential targets of PDAC, camptothecin, and sotorasib were 4432, 150, and 100, respectively as shown in Supplementary Material S2. A total of 32 (0.7%) commonly shared targets among PDAC, camptothecin, and sotorasib were identified, and their PPI networks are shown in Figure 11. Following this, several parameters such as MCC, degree, closeness, and betweenness were employed, and the top 10 hub genes such as TP53, HDAC1, BCL2, CASP1, mTOR, GSK3B, ATG5, EGFR, KRAS, and MAPK3 were identified. Their PPI network is shown in Figure 11. Notably, BCL2, CASP1, and TP53 are involved in apoptosis regulation; EGFR, KRAS, MAPK3, GSK3B, and mTOR are associated with the KRAS signaling pathway; ATG5 is involved in autophagy by regulating autophagosome formation; and HDAC1 is involved in epigenetic regulation. In addition, GO and KEGG pathway enrichment analyses were performed, and the enrichment terms, such as biological process (BP), cellular component (CC), molecular function (MF), and enriched KEGG pathways in cancer are shown in Figure 12. To note, the negative regulation of programmed cell death (GO:0043069), negative regulation of apoptosis (GO:0043066) and cellular response to starvation (GO:0009267) in BP enrichment; phagocytic vesicle (GO:0045335) and endocytic vesicle membrane (GO:0030666) in CC enrichment; ubiquitin protein ligase binding (GO:0031625) and kinase binding (GO:0019900) in MF enrichment are highly related to apoptosis and autophagy. Additionally, the KEGG-enriched pathway supported the involvement of the top 10 hub genes in apoptosis and autophagy, as shown in Figures 12, 13. From these results, we observed that camptothecin-induced DNA damage and elevated ROS levels lead to apoptosis induction and also suppresses autophagy by targeting ATG5 and LC3-II in PDAC.

## 4 Discussion

KRAS mutations are prevalent in several cancers such as PDAC, NSCLC, and CRC; and the G12, G13, and Q61 positions are frequently mutated hotspots (Burge and Hobbs, 2022; Luo et al., 2024). In comparison, KRAS G12C and KRAS G12D mutations are very common and pose a therapeutic challenge to target till 2020, after which it became druggable with the discovery of a selective, irreversible KRAS G12C inhibitor named sotorasib (AMG510) (Nagasaka et al., 2020; 2021; Conroy et al., 2021). Sotorasib potentially binds to the cysteine residue of the GDP-bound (OFF state) form of KRAS G12C, inhibits the downstream effectors of the MAPK pathway, and inhibits the formation of the GTP-bound (ON state) form of KRAS G12C, thereby limiting off-target effects (Lanman et al., 2020; Li R. et al., 2024). However, accumulating evidence indicates acquired resistance to sotorasib among patients in clinics (Ramalingam et al., 2023b; Kos and Saur, 2025). The CodeBreaK100 study (clinical trial information: NCT03600883) revealed that the objective response rate (ORR) was 41% and 12% for sotorasib-treated KRAS G12C-mutated NSCLC and KRAS G12C-mutated CRC respectively (Li et al., 2022). They also indicated that the combination of sotorasib and RTK upstream inhibitors could be a possible strategy to achieve a high therapeutic effect based on RTK pathway alterations in blood plasma levels. Another study indicated that sotorasib resistance is associated with the elevated expression of integrin β4 (ITGB4), and they reported that the ITGB4 and β-catenin inhibition enhanced the sotorasib effects (Mohanty et al., 2023).

To overcome sotorasib resistance, some combination studies, such as sotorasib with carboplatin and pemetrexed, sotorasib with metformin, sotorasib with adavosertib, sotorasib with DCC-3116, have been explored in preclinical studies, and most of the studies underlined the autophagy regulation and apoptosis-mediated cell death of KRAS-mutated cancers (Barrios-Bernal et al., 2023a; Ghazi et al., 2024; Li Q. et al., 2024; Yoshioka et al., 2024; Yamamoto et al., 2025). To note, a recent study reported that Avutometinib synergizes MRTX1133 and promoted apoptotic-mediated cell death in PDAC (Toyokuni et al., 2025). Natural compounds have been explored for their therapeutic potential against various cancers, including PDAC, NSCLC, and CRC (Hashem et al., 2022; Lou et al., 2024). Apoptosis is an important event in cancer treatment as it inhibits the proliferation, growth, and drug resistance of cancer cells. Autophagy is crucial for maintaining cellular homeostasis through cellular recycling (Pfeffer and Singh, 2018). Camptothecin is a natural alkaloid isolated from Camptotheca acuminata, which has been extensively reported for its significant potential to induce DNA damage, leading to apoptosis-mediated cell death in KRAS-mutated cancers (Don et al., 2024). Notably, camptothecin-induced apoptosis-mediated cell death has been reported in KRAS G12C-mutated MIA PaCa-2 and KRAS G12D-mutated PANC-1 PDAC cells (Fronza et al., 2011; Ragozin et al., 2018). Various reports have indicated that camptothecin, in combination with several anticancer compounds such as cisplatin, paclitaxel, gemcitabine, and docetaxel, significantly inhibits cancer cell proliferation by regulating apoptosis and autophagy in several cancers (Liu et al., 2024; Mitra et al., 2024; Odeniran et al., 2024). Based on these findings, we planned to study the synergistic potential of the apoptosis inducer camptothecin and the KRAS G12C inhibitor sotorasib in KRAS-mutated PDAC for the regulation of apoptosis and autophagy.

Earlier studies reported the IC_50_ of camptothecin in MIA PaCa-2 as 0.3–0.5 μM, and in PANC-1 as 0.18 μM at 48 h respectively (Fronza et al., 2011; Ragozin et al., 2018). Likewise, the reports indicated that the IC_50_ of sotorasib in MIA PaCa-2 was 34.1 nM, and in PANC-1 was >10 μM respectively (Chan et al., 2023; Fimm-Todt et al., 2023). From the experimentation, we found that the IC_50_ of sotorasib in MIA PaCa-2 and PANC-1 was determined as 0.04 μM and 20 μM, and the IC_50_ of camptothecin in MIA PaCa-2 and PANC-1 was determined as 0.5 μM and 0.3 μM respectively which are similar to the previous reports as shown in Figure 1, and camptothecin shows synergistic nature (CI < 1) towards sotorasib as shown in Figure 2. The combination index is widely used to measure the synergistic effects and pharmacodynamic interactions of multiple drugs for an efficient treatment (Sankar and Muthukaliannan, 2023). In the sotorasib + metformin combination study, the combination index for H23, A549, and H522 was CI = 0.62, 0.73, and 0.91, respectively. Notably, in all cells, they reported CI > 1, and comparatively, in A549 cells, the combination exerted significant anticancer potential owing to high Fa values.

Previous reports have indicated the potential of camptothecin and sotorasib to inhibit the proliferation, growth, colony formation, and migration of PDAC cells; however, their combinatorial effects have not been observed (Ragozin et al., 2018; Chan et al., 2023). In our study, we found that camptothecin + sotorasib combination has more potential than monotherapy (camptothecin and sotorasib alone) in inhibiting the proliferation, colony formation, and migration of KRAS-mutated PDAC cells. For instance, in the wound healing assay, the combination treatment resulted in 45% wound closure in MIA PaCa-2 cells and 42% wound closure in PANC-1 cells even after 48 h, whereas their monotherapy treatments showed a 1.5-to 2-fold increase in the migration of KRAS-mutated PDAC cells, as shown in Figure 3. Likewise, in the clonogenic assay, the combination treatment resulted in only 9% colony formation in MIA PaCa-2 and 10% colony formation in PANC-1, whereas their monotherapy treatments showed a 3-to 4-fold increase in colonies, as shown in Figure 4. In the present study, we observed that the combination exerted significant anti-migration effects and inhibited colony formation in cells.

Elevated ROS levels and DNA fragmentation are important factors associated in the regulation of apoptosis and autophagy (Chaturvedi et al., 2021). Increased ROS levels alter the expression of apoptotic- and autophagy-related genes and promote DNA fragmentation, which is usually observed in the late apoptotic stage, confirming cancer cell death without further growth (Zhdanov et al., 2020). A recent report indicated that a novel EGFR-targeting RNase A-cetuximab antibody-drug conjugate (RN-PEG-Cet) potentially induces DNA fragmentation and ROS-mediated cellular death in KRAS-mutant CRC (Jafary et al., 2025). Another interesting study reported a novel self-assembled nanoparticle (HA-TPP/A) that mediates DNA fragmentation and ROS-mediated apoptosis, and sensitizes KRAS-mutant PDAC and CRC cells to sotorasib. They also reported that this combination could be expanded to treat KRAS and TP53 co-mutations (Mei et al., 2024). Another study reported that the ULK1/2 inhibitor, DCC-3116, in combination with sotorasib, significantly reduced autophagic flux and showed synergy in KRAS-mutant NSCLC cells (Ghazi et al., 2024). In our study, we observed that the sotorasib + camptothecin combination showed a significant fold increase in ROS levels and in DNA fragmentation compared to monotherapies in KRAS-mutated PDAC cells. This indicates the significance of the combination therapy in possibly regulating apoptosis and autophagy in PDAC cells by elevating ROS levels and inducing DNA fragmentation as shown in Figure 5. Also, it is essential to mention that the relationship between the elevated ROS levels is only correlative and does not establish any direct relationship with autophagy. To better understand this relationship, future studies involving targeted ROS modulation will be important to clarify whether ROS actively contributes to the autophagic response.

From the flow cytometry analysis, we observed that the combination of sotorasib and camptothecin combination induced apoptosis in 88.61% and 86.66% of MIA PaCa-2 and PANC-1 cells, respectively, as shown in Figure 6. Notably, in monotherapy, camptothecin-treated cells induced more apoptosis than sotorasib, indicating a potent role of camptothecin in apoptosis-mediated cell death (Moreira et al., 2024). Additionally, the data strongly indicates that no considerable necrosis was not observed in any treatment group (combination or monotherapy), indicating apoptosis-mediated cell death (Zare-Mirakabadi et al., 2012). Camptothecin induced G2/M cell cycle arrest, whereas sotorasib and sotorasib + camptothecin induced G1/S cell cycle arrest. Notably, the combination treatment also increased the cell population in the Sub-G0/G1 phase, indicating apoptosis induction and DNA damage in the cells (Lai et al., 2022). Camptothecin treatment alone induced apoptosis, as observed in previous studies, due to its topoisomerase I inhibitory activity, which further promoted DNA fragmentation and apoptosis-mediated cell death (García et al., 2014). Additionally, LC3-II quantification indicated that sotorasib inhibited more LC3-II inhibition than camptothecin, whereas their combination showed reduced levels of LC3-II in both cell types, indicating the inhibition of autophagosome formation, a crucial step in autophagy, as shown in Figure 8 (Longobardi et al., 2024). Collectively, we observed that the combination treatment had a greater effect on cell cycle, apoptosis, and autophagy regulation than the monotherapies. However, as LC3-II accumulation alone does not clearly differentiate between increased autophagosome formation and impaired autophagic flux, we propose that future studies incorporate lysosomal inhibition assays and p62/SQSTM1 quantification to more accurately assess autophagic flux.

Apoptosis-mediated death of cancer cells is achieved by the upregulation of pro-apoptotic genes, such as BAX, BID, and BAK, and the downregulation of anti-apoptotic genes, such as BCL-2 and BCL-XL respectively (Saddam et al., 2024). In MIA PaCa-2 cells, the combination treatment significantly upregulated the pro-apoptotic genes BAX (3.5-fold increase), BID (3.8-fold increase), BAK (3.8-fold increase), and PARP (3-fold increase), and downregulated the pro-apoptotic genes BCL-2 (0.7-fold decrease) and BCL-XL (2-fold decrease). Similarly, in PANC-1 cells, the combination treatment significantly upregulated the pro-apoptotic genes BAX (3.5-fold increase), BID (3.5-fold increase), BAK (3.8-fold increase), and PARP (3.2-fold increase) and downregulated the pro-apoptotic genes BCL-2 (1.2-fold decrease) and BCL-XL (2.5-fold decrease). In the context of autophagy, combination treatment significantly downregulated the autophagy genes ATG5 (1.7-fold decrease) and MAP1LC3B (2.5-fold decrease) in MIA PaCa-2 cells, and ATG5 (2.1-fold decrease) and MAP1LC3B (2.8-fold decrease) in PANC-1 cells. Notably, in both the KRAS G12C-mutated and KRAS G12D-mutated cells, the sotorasib and camptothecin combination treatment significantly downregulated KRAS expression by 2.4-fold decrease and 2-fold, respectively, as depicted in Figures 9, 10 respectively. Overall, the heat map illustrating the fold changes in gene expression obtained from RT-PCR analysis was shown in Supplementary Figure S2 (Supplementary Material S1).

To further support the significance of camptothecin in the regulation of apoptosis and autophagy, we performed network pharmacology and enrichment analyses, as shown in Figures 11, 12 (Mishra et al., 2023c; 2023a). From the network pharmacology analysis, we observed that TP53, HDAC1, BCL2, CASP1, mTOR, GSK3B, ATG5, EGFR, KRAS, and MAPK3 were the top 10 hub genes that were common to all parameters, as shown in Table 2. Additionally, the GO- and KEGG-enriched pathways also supported the involvement of the top 10 hub genes in apoptosis and autophagy, as shown in Figure 13. Though, BCL-2 is involved in the anti-apoptotic process, it was enriched as a negative regulator of apoptosis (Pang et al., 2025; Vogler et al., 2025). However, we observed that camptothecin inhibits BCL-2 in both PDAC cell lines through qRT-PCR analysis, indicating its apoptosis-promoting activity. From these results, we observed that camptothecin-induced DNA damage and elevated ROS levels lead to apoptosis induction and also suppresses autophagy by targeting ATG5 and LC3-II in PDAC.

**TABLE 2:** Top 10 hub target proteins.

Gene name	Associated protein	UniProt ID	Mechanism related	Category
Apoptosis regulator Bcl-2	BCL2	P10415	Apoptosis	Protein Coding
Caspase-1	CASP1	P29466	Apoptosis	Protein Coding
Tumor protein p53	TP53	Q12888	Apoptosis	Protein Coding
Epidermal growth factor receptor erbB1	EGFR	P00533	MAPK pathway	Protein Coding
Kristen rat sarcoma viral oncogene homolog	KRAS	P01116	MAPK pathway	Protein Coding
Mitogen-activated protein kinase 3	MAPK3	P27361	MAPK pathway	Protein Coding
Autophagy Related 5	ATG5	Q9H1Y0	Autophagy	Protein Coding
Histone Deacetylase 1	HDAC1	Q13547	Epigenetic regulation	Protein Coding
Mechanistic Target Of Rapamycin Kinase	mTOR	P42345	Increased expression in KRAS mutated cancers	Protein Coding
Glycogen Synthase Kinase 3 Beta	GSK3β	P49841	Increased expression in KRAS mutated cancers	Protein Coding

Collectively, sotorasib inhibits KRAS signaling, impairs DNA damage, and maintains functional homologous recombination (HR) deficiency, whereas camptothecin maintains DNA damage by inhibiting topoisomerase activity (Chiou et al., 2023). Additionally, combination therapy showed greater potential than monotherapy for KRAS-mutated PDAC. From various analyses, such as intracellular ROS induction, DNA fragmentation, wound healing, colony formation, apoptosis detection, RT-PCR, and network pharmacology, we observed that the sotorasib and camptothecin combination treatment significantly suppressed autophagy and induced apoptosis-mediated cell death in both KRAS-mutated PDAC cell lines. In conclusion, we report the synergistic effect of the natural alkaloid camptothecin and the KRAS inhibitor, sotorasib, in KRAS-mutated cancer cells. Furthermore, we recommend examining more anti-cancer potential natural compounds to overcome clinical resistance in various cancers, including PDAC, CRC, and NSCLC (Chen et al., 2024; Jiang et al., 2024; Shao et al., 2024).

## 5 Conclusion

KRAS mutations are predominant in many cancers, including PDAC, NSCLC, and CRC, and alongside the FDA-approved KRAS G12C inhibitor, sotorasib, acquired clinical resistance. In our study, we investigated the synergistic potential of camptothecin and sotorasib in both KRAS G12C-mutated MIA PaCa-2 and KRAS G12D-mutated PANC-1 cells. From the drug synergy analysis, we predicted the synergism of camptothecin with sotorasib, and the combination treatment showed more potential in wound healing and colony formation assays than it is monotherapy. In addition, we found that the combination of camptothecin and sotorasib potentially induced intracellular ROS levels and DNA fragmentation, indicating that it could trigger apoptosis and regulate autophagy in PDAC cells. The apoptosis assay and cell cycle analysis showed that the combination has the potential to induce G1/S phase arrest with an increased Sub G0/G1 population, which leads to apoptosis-mediated cell death. Additionally, the combination treatment inhibited LC3-II levels, indicating the inhibition of autophagy. Furthermore, qRT-PCR analysis revealed that the combination significantly upregulated pro-apoptotic genes (BAX, BID, and BAK), downregulated KRAS pathway-related genes (KRAS, BRAF, MEK, and ERK), cleaved PARP, anti-apoptotic-related genes (BCL-2 and BCL-XL), and autophagy-related genes (ATG5 and MAP1LC3B) in both PDAC cells. Network pharmacology and enrichment analyses supported the significance of camptothecin in the regulation of apoptosis and autophagy. Collectively, we observed that the sotorasib exhibited significant potential in MIA PaCa-2 (KRAS G12C-mutated) than the PANC-1 (KRAS G12D-mutated) due to their KRAS G12C specificity. However, it also exhibited potential activity towards PANC-1 at higher concentrations and in combination with camptothecin, warranting that further studies are needed to evaluate the potential off-target or pathway-convergent mechanisms. Furthermore, the results have to be evaluated by protein expression assays and organoid and animal models for better understanding of the therapeutic potential of this combination. In conclusion, we report the synergistic effect of the natural alkaloid camptothecin and the KRAS inhibitor, sotorasib, in KRAS-mutated cancer cells. Furthermore, we recommend examining more anti-cancer potential natural compounds to overcome the clinical resistance of approved anti-cancer drugs such as sotorasib in the near future.

## Data Availability

The original contributions presented in the study are included in the article/Supplementary Material, further inquiries can be directed to the corresponding authors.
