# Supplementary material for: Synergistic anticancer effects of camptothecin and sotorasib in KRAS-mutated pancreatic ductal adenocarcinoma
Source: Front Pharmacol. 2025 Jul 18;16:1635449. doi: 10.3389/fphar.2025.1635449 (PMC12314289; doi:10.3389/fphar.2025.1635449)
Supplement: Supplementary file 1 [file Supplementaryfile1.docx]

Synergistic Anticancer Effects of Camptothecin and Sotorasib in

*KRAS*-Mutated Pancreatic Ductal Adenocarcinoma

Prasanna Srinivasan Ramalingam^1^, Gayathri Chellasamy^2^, Md Sadique Hussain^3,4^, Kodiveri Muthukaliannan Gothandam^5^, Tajamul Hussain^6,7^, Salman Alrokayan^7^, Kyusik Yun^2^, Janaki Ramaiah Mekala^8,*^, Sivakumar Arumugam^1,**^

^1^Protein Engineering lab, School of Biosciences and Technology, Vellore Institute of Technology, Vellore, Tamil Nadu, India

^2^Department of Bionanotechnology, Gachon University, Gyeonggi-do, Republic of Korea

^3^Uttaranchal Institute of Pharmaceutical Sciences, Uttaranchal University, Dehradun, Uttarakhand 248007, India

^4^School of Pharmaceutical Sciences, Lovely Professional University, Phagwara 144411, Punjab, India.

^5^High Throughput Lab, School of Biosciences and Technology, Vellore Institute of Technology, Vellore, India

^6,7^Centre of Excellence in Biotechnology Research, King Saud University, Riyadh 11451, Saudi Arabia

^7^Research Chair for Biomedical Application of Nanomaterials. Biochemistry Department, College of Science, King Saud University, Riyadh 11451, Saudi Arabia

^8^School of Biosciences and Technology, Vellore Institute of Technology, Vellore, India

*Corresponding author (main): Janaki Ramaiah Mekala ([janakiramaiah.m@vit.ac.in](mailto:janakiramaiah.m@vit.ac.in))

**Corresponding author: Sivakumar Arumugam ([siva_kumar.a@vit.ac.in](mailto:siva_kumar.a@vit.ac.in))


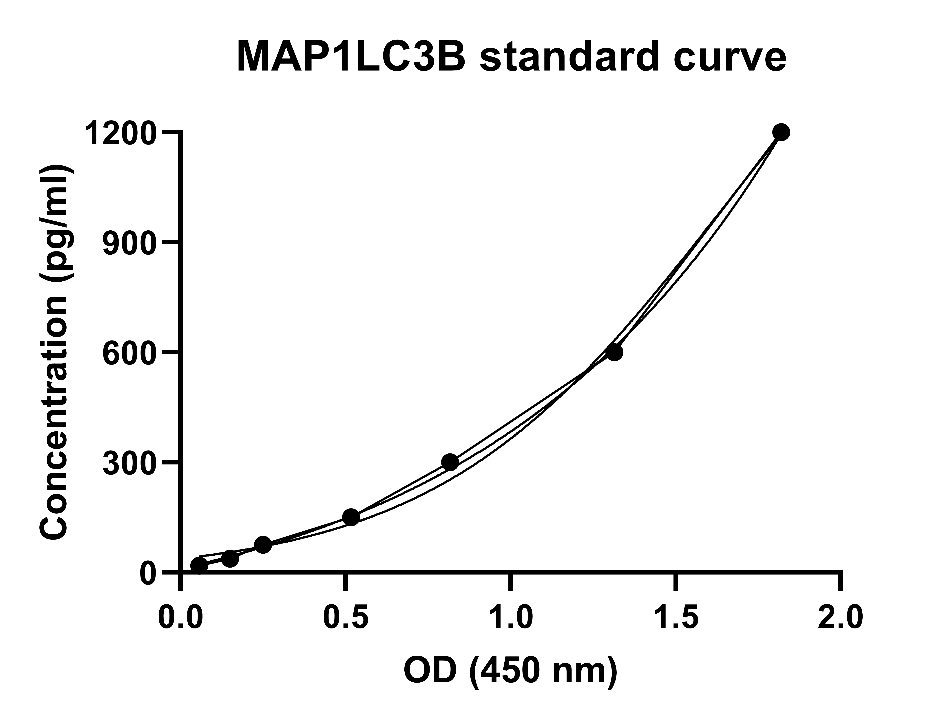


**Supplementary Figure 1:** Standard curve of MAP1LC3B used for interpolation of samples


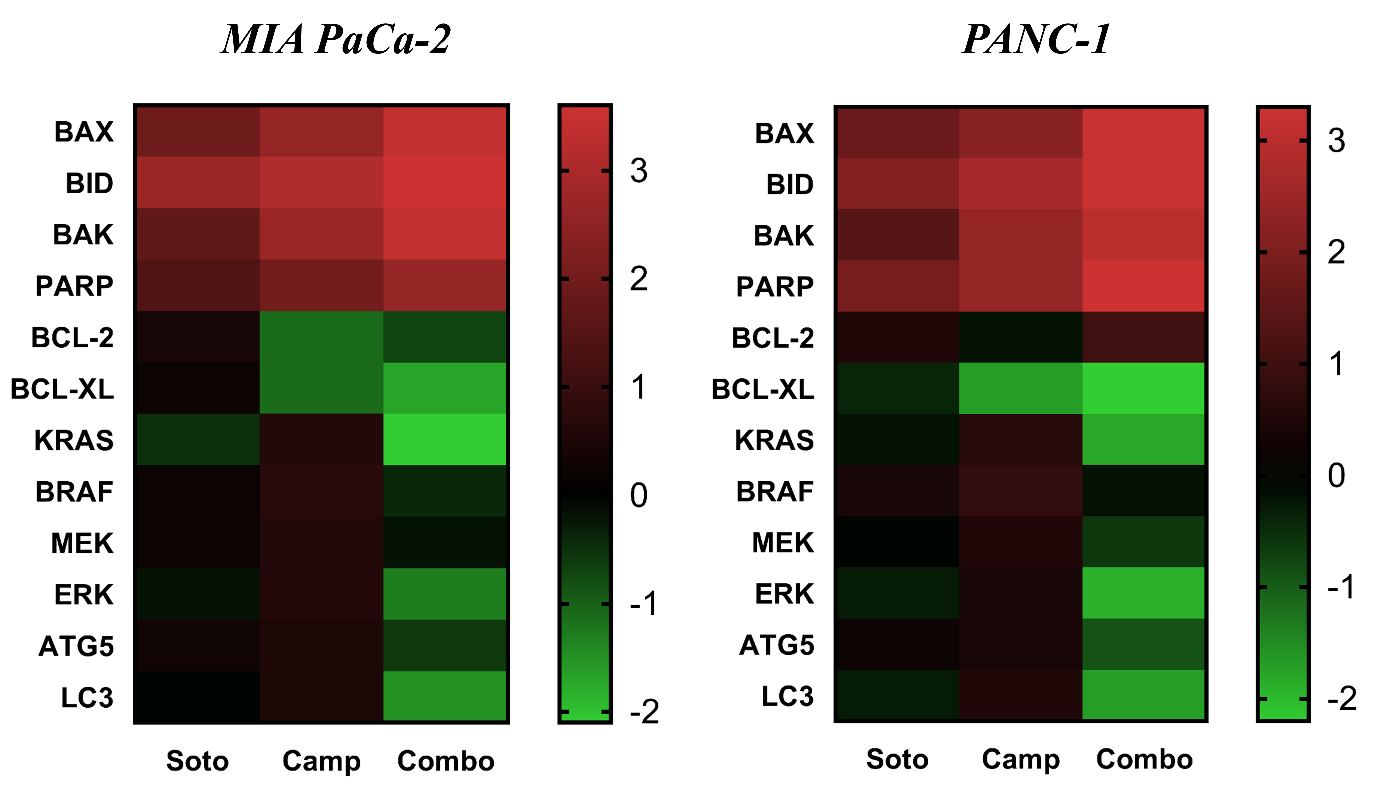
**Supplementary Figure 2:** Heat map illustrating the fold changes in gene expression obtained from RT-PCR analysis in MIA PaCa-2 and PANC-1 cells
